# Diminished expression of major histocompatibility complex facilitates the use of human induced pluripotent stem cells in monkey

**DOI:** 10.1186/s13287-020-01847-9

**Published:** 2020-08-03

**Authors:** Xiaokai Wang, Meng Lu, Xiaoyu Tian, Yansong Ren, Yijun Li, Meng Xiang, Sifeng Chen

**Affiliations:** Department of Physiology and Pathophysiology, School of Basic Medical Sciences, Fudan University, Shanghai, 200032 People’s Republic of China

**Keywords:** Induced pluripotent stem cells, Major histocompatibility complex, Stem cell immunogenicity, Non-human primate, Wound healing

## Abstract

**Background:**

Stem cells, including induced pluripotent stem cells (iPSCs), have tremendous potential in health care, though with several significant limitations. Each of the limitations, including immunogenicity, may block most of the therapeutic potentials. Beta2 microglobulin (B2M) and MHC II transactivator (CIITA) are critical for MHC I and II, respectively. MHCs are responsible for immunogenic recognition.

**Methods:**

B2M and CIITA were knocked out from human iPSCs, either separately or simultaneously. The effects of single or dual knockout of B2M and CIITA on iPSC properties were evaluated in a xenogeneic model of human-to-monkey transplantation.

**Results:**

B2M or CIITA knockout in human induced pluripotent stem cells (iPSCs) diminishes the expression of MHC I or II alleles, respectively, without changing iPSC pluripotency. Dual knockout was better than either single knockout in preserving the ability of human iPSCs to reduce infiltration of T and B lymphocytes, survive, and promote wound healing in monkey wound lesions. The knockouts did not affect the xenogeneic iPSC-induced infiltration of macrophages and natural killer cells. They, however, decreased the iPSC-promoted proliferation of allogeneic peripheral blood mononuclear cells and T lymphocytes in vitro, although not so for B lymphocytes isolated from healthy human donors. Although the dual knockout cells survived long enough for suiting therapeutic needs, the cells eventually died, possibly due to innate immune response against them, thereby eliminating long-term risks.

**Conclusions:**

Having these iPSCs with diminished immunogenicity-recognizable to allogeneic recipient may provide unlimited reproducible, universal, standardized “ready-to-use” iPSCs and relevant derivatives for clinical applications.

## Highlight

Genetically, ablation of MHC complexes reduces the immunogenicity and increases the survival, safety, and therapeutic potential of iPSCs in vivo. Having iPSCs with immune recognition between autologous and allogeneic stem cells may provide universal, standardized “ready-to-use” iPSCs and relevant derivatives for clinical applications.

## Background

Patient safety is a priority in effective stem cell therapy. The bottleneck issues challenging stem cell application include unwanted differentiation [1, 2], stem cell thrombosis [3–5], transplant-recipient immunogenicity [6, 7], and integration failure [8]. Besides, there is a dilemma regarding the time, workload, and economic burden of individualized autologous iPSC generation and immune rejection of allogeneic iPSCs and their derivatives. Solving these problems, one by one, would eventually lead to the widespread application of stem cell therapy in real clinical scenario.

Eventual death of stem cells after completing their therapeutic goals provides additional safety in preventing unwanted differentiation or proliferation of in vivo administered stem cells. Most cell types in the body are not eternal, namely epithelial [9], endothelial [10, 11], and epidermal cells [12]. We found intravenously administered iPSCs trafficked to acute injured lungs, differentiated into epithelial cells, and decreased lung injury. The survival time of the cells in the injured lungs was between 28 and 90 days. Stem cells and their differentiated types or subcellular products (such as exosomes) may provide a temporary replacement of damaged cells, prevent deformation due to the missing parenchymal cells, create microenvironment or scaffold for internal regeneration, rejuvenate aging cells, promote angiogenesis, or simply transplant engineered tissue. For example, we found iPSC-derived endothelial progenitor cells have the same therapeutic effect as iPSCs along with an in vivo half-life similar to endothelial cells [13]. Bedsides, immune response induces by dead stem cell may benefit tissue injury [14]. Thus, using reduced immune-recognizable allogeneic cells or their derivatives will not only eliminate lengthy, expensive, and unstandardized autologous iPSC induction and subsequent differentiation into linkage-specific cells or tissue, but also provide additional safety.

Beta2 microglobulin (B2M) is part of the major histocompatibility complex (MHC) class I complex and MHC II transactivator (CIITA) is critical for MHC II production [15]. MHCs (also known as “HLAs” in humans) are responsible for immunogenic recognition. Deuse et al. have shown that hypoimmunogenic derivatives of induced pluripotent stem cells that overexpress CD47 evade immune rejection in fully immunocompetent allogeneic recipients [16]. Single administration of such hypoimmunogenic iPSCs and their derivatives evaded immune rejection and survived for long, as teratoma and cell plugs, in allogeneic recipients, without immunosuppression. While elimination of both innate and adaptive responses may be necessary for supplying eternal cells, such as neurons and muscle cells, complete elimination of NK cell-mediated innate immune response may not be preferable with respect to long-term risks. Diminishing adaptive immune response may in fact reduce the speed of natural killing, immune-induced inflammation [17] and adhesion molecule expression in endothelial cells required for NK cell infiltration [18, 19].

In this study, the effects of single or dual knockout of B2M and CIITA on iPSC properties were evaluated in a xenogeneic model of human-to-monkey transplantation, considering monkeys to be closer to humans in various perspectives. Besides, whether the immune response and iPSC microenvironment in humanized mouse would fully represent those in humans remains unknown, since the tissue microenvironment and cytokine profile required for immune development are different between humans and mice.

## Methods

### Animals

The animal protocol was approved by the Animal Care Committee of the Fudan University Shanghai Medical College in accordance with the Guide for the Care and Use of Laboratory Animals (National Research Council of the USA). All procedures involving animals were performed in accordance with institutional guidelines and permission of the Ethics Committee at the Fudan University Shanghai Medical College. Seven adult male rhesus macaques from unrelated consanguineous families (all were farm-raised 3rd generation) were purchased from the Dongwu Experimental Monkey Farm (Ningbo, China) and Jingde Monkey Professional Cooperation (Anhui, China). They were housed in an animal laboratory at the Fudan University-affiliated Shanghai Public Health Clinical Centre (Shanghai, China). As a prerequisite to enter the housing facility, they were tested for simian immunodeficiency virus, simian type D retrovirus, simian T lymphotropic virus, herpes B virus, and tubercle bacillus; none of these viruses was detected. The animals were 16–18-year-old virgins.

### Generation and culture of human iPSCs

Human iPSCs were generated, cultured, and characterized as we previously described [20]. Briefly, peripheral blood nucleated cells were isolated and infected with Sendai viruses carrying a polycistronic construct of human *Klf4*, *Oct3/4*, *Sox2*, and *c-Myc*. Cells were cultured on dishes coated with Matrigel diluted at 60: 1 (BD Biosciences) in mTeSR1 medium (STEMCELL Technologies, Canada). iPSC clones were amplified and then characterized by assessing the expression of pluripotency markers and through in vitro differentiation, alkaline phosphatase activity, and teratoma formation assays, in addition to karyotyping [20].

### Generation of B2M-knockout, CIITA-knockout, B2M and CIITA-knockout, and HLA-A-knockout iPS cell lines

B2M, CIITA, and HLA-A CRISPR were designed using the Zhang lab’s protocol [21] and MIT’s CRISPR design software (http://crispr.mit.edu). Oligonucleotides (IDT) corresponding to B2M, CIITA, and HLA-A were as follows:

**Table:** 

	Sequences (5′-3′)	Antisequences (5′-3′)
B2M	CACCGAAGTTGACTTACTGAAGAA	AAACTTCTTCAGTAAGTCAACTTC
CIITA	CACCGCCTGGCTCCACGCCCTGCT	AAACAGCAGGGCGTGGAGCCAGGC
HLA-A	CACCGAGGGTTCGGGGCGCCATGA	AAACTCATGGCGCCCCGAACCCTC

SgRNAs were annealed and ligated to the Cas9 nickase-puromycin plasmid pX462 (Addgene Plasmid #62987, Cambridge, MA) that had been digested with BbsI to generate the individual guide plasmid. For electroporation, 1 × 10^5^ cells were re-suspended in R Buffer of the electroporation kit, mixed with 6 μg of guide plasmid DNA, and electroporated at 1100 V for one 10 ms pulse, using a 10 μL Neon Transfection System (MPK1096, Life Technologies, Carlsbad, CA). From these transfected cells, single-cell clones were generated by serial dilution in 100-mm dishes. Successful B2M, CIITA, and HLA-A knockout were confirmed by RT-PCR and Western blotting. Genomic DNA was isolated from the cloned cells using QuickExtract-DNA Extraction Solution (Lucigen Co., Madison, WI) and prepared for sequence analysis using the pEASY-Blunt Zero Cloning Kit (Civic Bioscience Ltd., Beloeil, QC, Canada). DNA was PCR amplified using the following primers:

**Table:** 

Primers	Sequences (5′-3′)	Antisequences (5′-3′)
B2M	GGGAAGGTGGAAGCTCATTT	TGGGACTCATTCAGGGTAGTAT
CIITA	GGTTAGTGATGAGGCTAGTGATG	AGAACCTTTCGGTGCTGATAC
HLA-A	CAAGACTCAGGGAGACATTGAG	GGGACACGGATGTGAAGAAATA

DNA sequencing analysis of the cloned products confirmed the generation of indels at the targeted genomic site of one clone. The cloned cell lines were named as B2M^−/−^-iPSCs, CIITA^−/−^-iPSCs, and HLA-A^−/−^-iPSCs. Repeated knockout of CIITA in the B2M^−/−^-iPSCs cell line was performed to obtain B2M^−/−^ and CIITA^−/−^-iPSCs.

### Real-time PCR and Western blotting of knockout and MHC-related genes

Total RNA and protein were extracted from cultured iPSCs using TRIzol Reagent (Thermo Fisher Scientific, Waltham, MA) and RAPI buffer (Thermo Fisher), respectively. RNA concentrations and purities were determined using a NanoDrop instrument (Thermo Fisher) at wavelengths of 260/280 nm. cDNA was synthesized from 1 μg of total RNA using SuperScript III (Thermo Fisher) according to the manufacturer’s instructions. The cDNA was diluted with DNase-free water to a concentration of 10 ng/μl. Real-time PCR was performed in a Bio-Rad iQ Real-Time PCR Detection System using the IQ SYBR Green Supermix (Bio-Rad, Hercules, CA) with the following thermal cycle program: initial denaturation at 95 °C for 5 min, followed by 40 cycles of 95 °C for 20 s, 57 °C for 45 s, and 72 °C for 30 s. The mRNA levels of target genes were normalized to β-actin, as the internal control, and expressed relative to the quantity of the control group. The cDNA was PCR amplified using the following primers:

**Table:** 

Primers	Sequences (5′-3′)	Antisequences (5′-3′)
B2M	GAGGCTATCCAGCGTACTCCA	CGGCAGGCATACTCATCTTTT
CIITA	CTGGCTGGAGAAGAAGAGATTG	AGTTCCGCGATATTGGCATAA
HLA-A	GTGGACGACACGCAGTT	TGTGAGTGGGCCTTCATATTC
HLA-B	GTATTTCTACACCTCCGTGTCC	CTGTCGAACCTCACGAACTG
HLA-C	TACTACAACCAGAGCGAGGA	CTCGTTCAGGGCGATGTAAT
HLA-DRA	ATCATCCAGGCCGAGTTCTA	CCGTCTCCTTCTTTGCCATATC
HLA-DRB	GACAACTACTGCAGACACAACTA	CAGAACAGACCAGGAGGTTATG
HLA-DQA	CAACACCCTCATTTGTCTTGTG	CTCAGAAACACCTTCTGTGACT
HLA-DQB	GGTAGCAACTGTCACCTTGAT	GTGAAGTAGCACATGCCCTTA
HLA-DPA	CCCTGAAGACAGAATGTTCCATA	CAAACGCGGCATAAGTTGAC
HLA-DPB	AGGGCCACTCCAGAGAATTA	CTCCTCCCGGTTGTAGATGTAT
β-Actin	GGACCTGACTGACTACCTCAT	CGTAGCACAGCTTCTCCTTAAT

Protein lysates were separated by sodium dodecyl sulfate-polyacrylamide gel electrophoresis (10% resolving gel) and transferred to a polyvinylidene difluoride membrane. The membrane was blocked and then incubated with rat monoclonal anti-CD3 antibody (Abcam Inc., Cambridge, UK), rabbit monoclonal anti-CD20 antibody (Bioworld Technology Inc., St. Louis Park, MN), mouse monoclonal anti- NCAM-1/CD56 antibody (R&D Systems, Inc., Minneapolis, MN), or mouse monoclonal anti-CD68/SR-D1 antibody (Novus Biologicals Inc., Centennial, CO), followed by Peroxidase AffiniPure Donkey Anti-rat, Anti-rabbit, or Anti-mouse IgG (H + L) (Jackson ImmunoResearch Inc., West Grove, PA) as the secondary antibodies, respectively. The immunoreactive bands were visualized using enhanced chemiluminescence substrate (Thermo Fisher).

### Detection of cells in the wound lesion

To identify the transplanted cells in the wound lesion, one of the housekeeping genes, HSP90, whose consistency rate between human and monkey sequence was 84.39%, was selected as a detection index. Total RNA was isolated from wound skin tissue using TRIzol reagent (Thermo Fisher), as described previously [6], and primers were designed according to the human-specific sequences (the first 365 bp). The cDNA was PCR-amplified using the following primers:

**Table:** 

Primers	Sequences (5′-3′)	Antisequences (5′-3′)
HSP90	CCACTCTACTCTGTCTCTGGAA	GCAGATCCTTGTAGAGGTGTTG
β-Actin	GGACCTGACTGACTACCTCAT	CGTAGCACAGCTTCTCCTTAAT

### Alkaline phosphatase detection

Alkaline phosphatase (AP) staining was performed using an AP Detection Kit (Sigma-Aldrich, St. Louis, MO) according to the manufacturer’s instructions.

### Embryoid body formation assay

The in vitro differentiation ability of iPSC lines was analyzed by embryoid body (EB) formation. Undifferentiated iPSC colonies were transferred into gelatin (0.2%)-coated petri dish and cultured for spontaneous differentiation in EB medium for 7 days. Theoretically, the resulting EB comprised of three embryonic germ layers.

To prepare 500 ml of human iPSC embryoid body medium, 387.5 ml of Dulbecco’s modified Eagle’s medium/nutrient mixture F-12 medium (DMEM/F12, Thermo Fisher) was mixed with 100 ml of KnockOut Serum Replacement (KSR, Thermo Fisher), 5 ml of GlutaMAX Supplement (1 mM, Thermo Fisher), 5 ml of penicillin-streptomycin (Thermo Fisher), 5 ml of Non-Essential Amino Acids Solution (NEAA, Thermo Fisher), and 500 μl of 2-mercaptoethanol (100 mΜ, 2-ME, Thermo Fisher). The medium was filtered using a 0.22-μm filtration unit and stored for up to a week at 4 °C.

### Immunofluorescence of pluripotency markers

Cells were fixed in 4% paraformaldehyde at room temperature for 20 min, rinsed with PBS, and subsequently blocked by 5% donkey serum (Jackson ImmunoResearch Inc.) at room temperature for 60 min. For cytoplasmic protein staining, 0.3% Triton X-100 was added for permeabilization. Cells were then incubated with primary antibodies diluted in 5% donkey serum, mouse monoclonal anti-Oct3/4 antibody (Abcam Inc.), rabbit polyclonal anti-SOX2 antibody (Abcam Inc.), and mouse monoclonal anti-SSEA4 antibody (Abcam Inc.), respectively, at 4 °C overnight. Cells were washed and exposed to secondary antibodies at room temperature for 60 min. Their nuclei were finally counter-stained with 1 μg/ml blue fluorescent dye, 4′, 6-diamidino-2-phenylindole, dihydrochloride (DAPI, Thermo Fisher).

### Cell viability assay

iPSCs were seeded in a 96-well plate in culture medium for 24 h to determine cell proliferation. Cell Counting Kit-8 (Dojindo Molecular Technologies, Gaithersburg, MA) was used according to the manufacturer’s instructions. Absorbance was measured at 450 nm using a spectrophotometer/microplate reader (BioTek, Winooski, VT).

### Cell apoptosis analysis

Flow cytometry analysis of apoptotic proteins (Anti-Annexin V-FITC/PI, BD Pharmingen, San Jose, CA) was performed with a FACSCalibur™ flow cytometer (BD Biosciences). An isotype control matching the immunoglobulin subtype was used to stain the cells analogously to control non-specific binding. The data were analyzed using FlowJo software (FlowJo Inc., Ashburn, OR).

### Teratoma formation in C57BL/6 mice and monkeys

To confirm their ability to form teratomas, 5 × 10^6^ iPSCs, in a volume of 100 μl (50 μl medium and 50 μl Matrigel), were subcutaneously injected into C57BL/6 mice and monkeys. Sequential teratoma generation was observed, and the injected cells along with surrounding tissue were harvested to examine the immune response.

### Full-thickness excisional skin wound model in monkeys

For major surgeries, the animals were injected with atropine sulfate (0.05 mg/kg). After 30 min, the monkeys were anesthetized by intra-muscular injection of an anesthetic mixture (0.15 ml/kg body weight). Each milliliter of the mixture contained 33.33 mg ketamine hydrochloride, 40 mg xylazine, 2.67 μg dihydroetorphine, and 1.67 mg haloperidol. Macaques were anesthetized before creating full-thickness excision wounds. The macaque dorsal skin was prepared by removing hair with a depilatory cream. Full-thickness excision wounds were created by excising the full-thickness skin in the mid-back with a 4-mm Biopunch (Fray Products Corp., Buffalo, NY).

### Wound closure evaluation

Images of the wounds were captured on days 0, 3, 6, 9, and 12. Wound areas were quantitated by the ImageJ software. Wound closure was calculated using the following equation: wound closure (%) = (wound area on day 0 − wound area on the indicated day) × 100%/wound area on day 0.

### Detection of antibodies against administered human iPSCs in monkey serum

The enzyme-linked immunosorbent assay (ELISA) was used to detect and quantitate the concentration of antibody in serum samples from monkeys that received all of WT, B2M^−/−^, CIITA^−/−^, and B2M^−/−^ and CIITA^−/−^ iPSCs. Briefly, 2 × 10^4^ iPSCs/well were seeded onto the 96-well plate and cultured for 4 h. Each of WT, B2M^−/−^, CIITA^−/−^, and B2M^−/−^ and CIITA^−/−^ iPSCs was contained in 16 wells (Supplemental Table 1). All cells were then fixed with 4% paraformaldehyde plus 1% methanol at room temperature for 5 min and rinsed with PBS. Cells in half of the wells were treated with 0.3% Triton X-100 for 5 min to permeabilize the cell membrane for staining of intracellular antigens. Wells were arranged as shown in the supplemental table 1. All wells were blocked with 5% donkey serum at room temperature for 45 min, followed by incubation with monkey serum diluted in 5% donkey serum (1:50) at 37 °C for 1 h. Cells were washed and exposed to secondary antibody, Peroxidase Affinipure Donkey Anti-human IgG (H + L)(1:5000, Jackson ImmunoResearch Inc., West Grove, PA), at 37 °C for 45 min. The cells were finally washed and allowed to react with 3,3′,5,5′-tetramethylbenzidine (TMB, Merck KGaA, Darmstadt, Germany) for 5 min at room temperature. The reaction was terminated with stop solution (Merck KGaA, Darmstadt, Germany). Optical density (OD) at 450 nm was measured with a spectrophotometer/microplate reader (BioTek).

### Co-culture of peripheral blood immune cells and iPSCs

Peripheral blood mononuclear cells (PBMCs) were prepared from 5 healthy donors (male, age between 23 and 32 years). CD3- and CD19-positive lymphocytes were prepared separately from the PBMCs using flow cytometry (BD Biosciences) after being labeled with primary antibodies carrying red or green fluorescence, respectively. They were then co-cultured with different iPSC lines, respectively.

For PBMC and iPSC co-culture, 5 × 10^5^ iPSCs/well were labeled with PKH26 red fluorescent cell linker (Sigma-Aldrich) and seeded in the 6-well plate. Four hours after iPSC seeding, 1 × 10^6^ PBMCs were labeled with PKH67 green fluorescent cell linker (Sigma-Aldrich) and seeded onto the iPSC surfaces in the wells.

For CD3^+^ lymphocyte and iPSC co-culture, 5 × 10^5^ iPSCs/well were labeled with PKH67 green fluorescent cell linker (Sigma-Aldrich), and 1 × 10^6^ CD3^+^ lymphocytes were seeded onto the iPSC surfaces in the wells.

For CD19^+^ lymphocyte and iPSC co-culture, 5 × 10^5^ iPSCs/well were labeled with PKH26 red fluorescent cell linker (Sigma-Aldrich), and 2 × 10^4^ CD19^+^ lymphocytes were seeded onto the iPSC surfaces in the wells.

After incubation for 7 days, the total number of cells in each well was counted. The CD3^+^ lymphocyte and iPSC co-cultures were washed and labeled with PE anti-human CD3 (Sony Biotechnology Inc., San Jose, CA), for 30 min at room temperature. CD19^+^ lymphocyte and iPSC co-cultures were washed and labeled with fluorescein-label monoclonal mouse-CD19 antibodies (Abcam Inc., ab24936) for 30 min. All samples were analyzed using a FACSCalibur™ flow cytometer (BD Biosciences). Data were analyzed using FlowJo software (version 10.).

### Statistical analysis

The results are presented as mean ± standard deviation (mean ± SD). Paired, unpaired, or chi-square test was used for statistical comparison of the data, as described in the figure legends. Differences between groups were considered significant when *P* < 0.05.

## Results

### Confirmation of beta2 microglobulin and major histocompatibility complex class II transactivator knockout

**Fig. 1:**
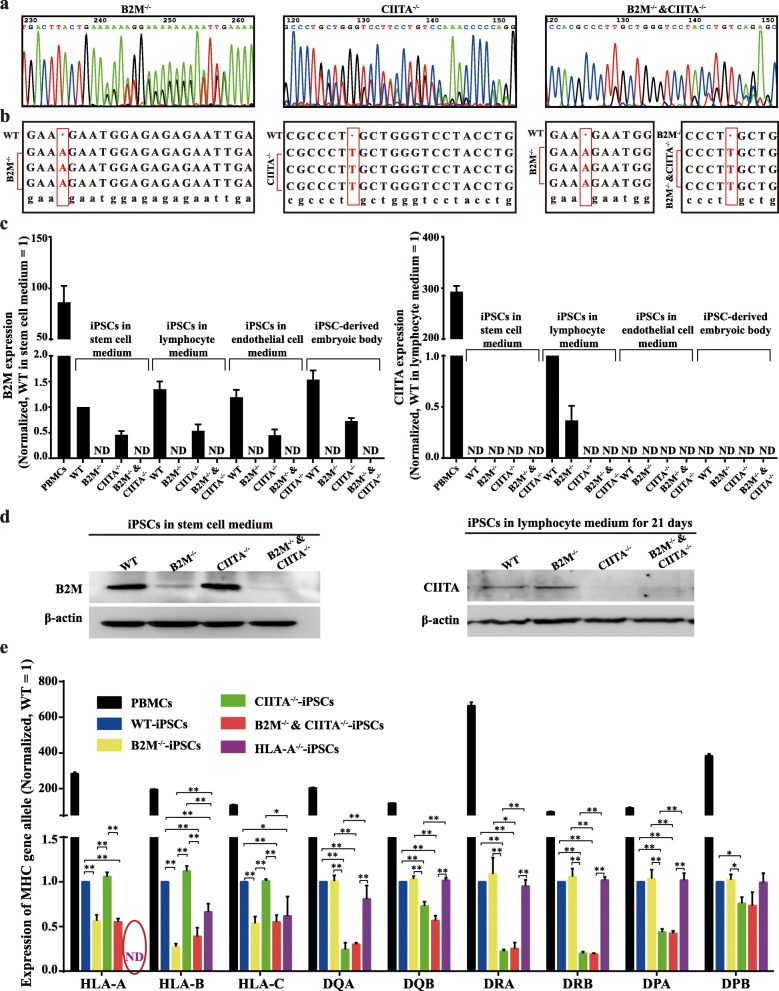
Generation and characterization of knockout iPSCs and expression of genetic alleles of major histocompatibility complex I and II in different iPS cell lines. **a** DNA sequencing results of different cell lines showed the double peak phenomenon and **b** T-vector test showed the deletion loci. **c** Real-time PCR demonstrated B2M knocked out in B2M^−/−^-iPSCs and B2M^−/−^ and CIITA^−/−^-iPSCs as well as in their derivatives. CIITA was knocked out in CIITA^−/−^-iPSCs and B2M^−/−^ and CIITA^−/−^-iPSCs as well as in their derivatives. **d** Western blot analysis showed B2M and CIITA expression in different iPS cell lines cultured in stem cell and lymphocyte medium, respectively. **e** PCR detection of alleles of the major histocompatibility complex I and II in peripheral blood mononucleated cells, WT-iPSCs, B2M^−/−^-iPSCs, HLA-A^−/−^-iPSCs, CIITA^−/−^-iPSCs, and B2M^−/−^ and CIITA^−/−^-iPSCs. Data are representative of three repeats

**Fig. 2:**
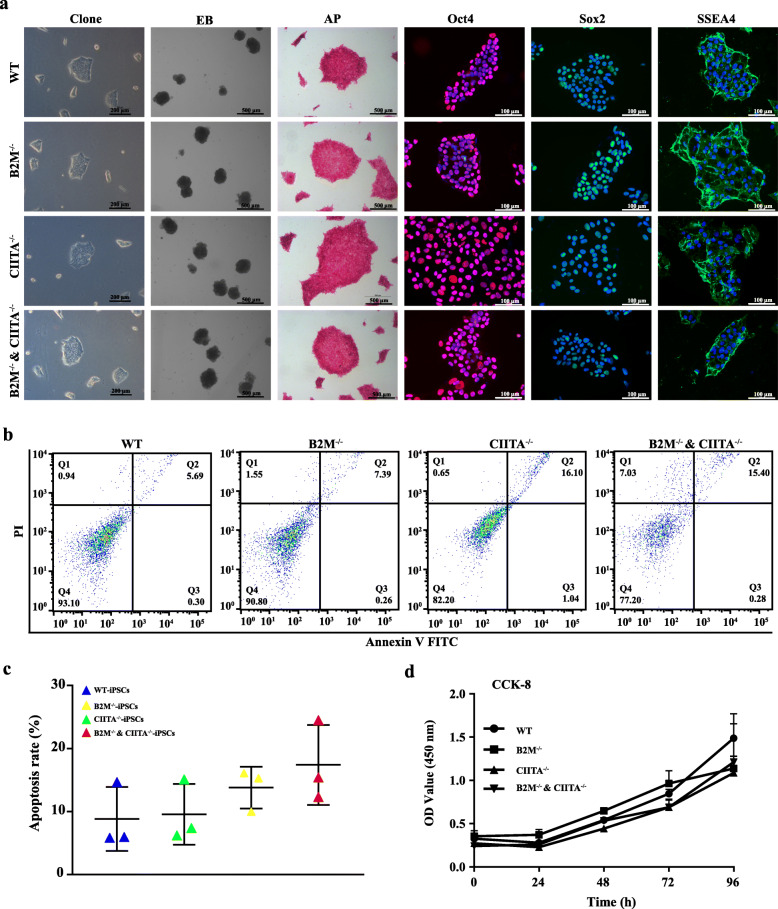
Biological characteristics of knockout iPSCs. **a** WT-iPSCs, B2M^−/−^-iPSCs, CIITA^−/−^-iPSCs, and B2M^−/−^ and CIITA^−/−^-iPSCs had normal clone morphology, were capable of forming embryonic body, had alkaline phosphatase activity, and were positive for Oct3/4, Sox2, and SSEA4. **b**, **c** Knockout of B2M and/or CIITA did not affect iPSC apoptosis. **d** Knockout of B2M and/or CIITA did not affect iPSC proliferation. Data represent three independent repeats

Human iPSCs were generated and identified as we described previously [20]. B2M and/or CIITA knockout by CRISPR-Cas9 was confirmed by DNA sequencing of the 3 human iPSC cell lines (B2M^−/−^, CIITA^−/−^, and B2M^−/−^ and CIITA^−/−^) showing the double peak phenomenon (Fig. 1 a), and T-vector test showing the deletion loci (Fig. 1 b). B2M mRNA and protein were absent in B2M^−/−^ and B2M^−/−^ and CIITA^−/−^ but not in WT or CIITA^−/−^, including iPSCs, iPSCs cultured in lymphocyte for 21 days or in endothelial cell medium for 7 days, and iPSC-derived embryonic body (Fig. 1c, d). CIITA mRNA and protein were present in B2M^−/−^, but not in CIITA^−/−^ or B2M^−/−^ and CIITA^−/−^ cultured in lymphocyte medium for 21 days. As expected, all the cell lines and their derivatives cultured in a medium other than lymphocyte medium did not express CIITA (Fig. 1c, d). As a positive control, peripheral blood mononucleated cells (PBMCs) were positive for both B2M and CIITA (Fig. 1c). The general biological characteristics, including clone and embryonic formations, growth, and apoptosis, as well as expressing pluripotent markers, were not significantly different across the four cell lines (Fig. 2).

### B2M and/or CIITA knockout reduces the expression of MHC I and/or II alleles

**Fig. 3:**
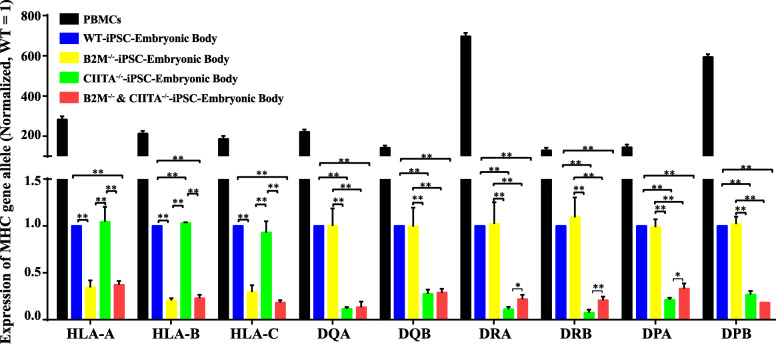
Expression of alleles of major histocompatibility complex I and II in different iPSC-embryonic bodies. Expression of alleles of the major histocompatibility complex I and II in different iPSC-embryonic bodies

**Fig. 4:**
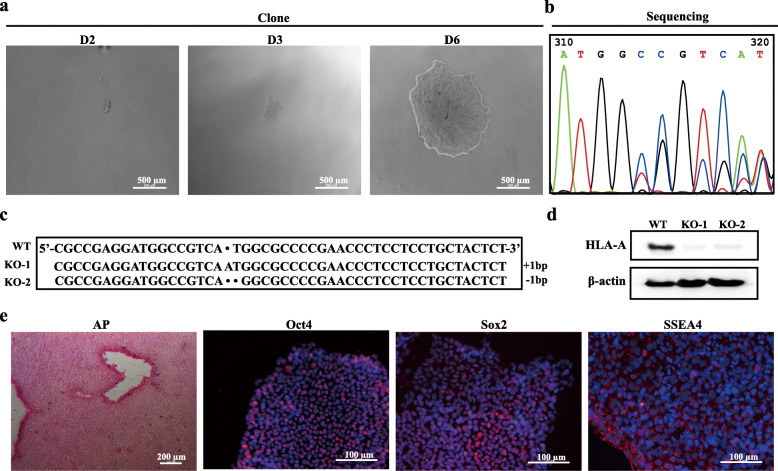
Generation and characterization of HLA-A knockout iPSCs. **a** Clone morphology after electroporation. **b** DNA sequencing results of knockout cell lines showed the double peak phenomenon. **c** DNA sequencing results showed the deletion loci. **d** Western blot analysis revealed HLA-A knocked out in iPSCs. **e** HLA-A^−/−^-iPSCs had alkaline phosphatase activity and were positive for Oct3/4, Sox2, and SSEA4

Single knockout of B2M diminished the expression of genetic alleles of MHC I (HLA-A, HLA-B, and HLA-C), whereas that of CIITA diminished the expression of genetic alleles of MHC II (DQA, DQB, DRA, DRB, DPA, and DPB). Simultaneous knockouts of B2M and CIITA diminished the expression of all MHC I and II genetic alleles (Fig. 1e). The result of iPSCs and iPSC-derived embryonic bodies was similar (Fig. 1e and Fig. 3), although all alleles were still detectable. To exclude the possibility of a lack of specificity of the PCR primers causing the expression to remain detectable, HLA-A was knocked out successfully using CRISPR-Cas9, followed by measurement of general biological characteristics (Fig. 4). PCR showed HLA-A to be completely non-detectable in HLA-A knockout iPSCs (Fig. 1e). This result indicated that the knockout of B2M did not completely block the expression of corresponding MHC I genetic alleles, at least in the case of HLA-A.

### Both B2M and CIITA knockout contribute to the teratogenicity of human iPSCs in monkeys

**Fig. 5:**
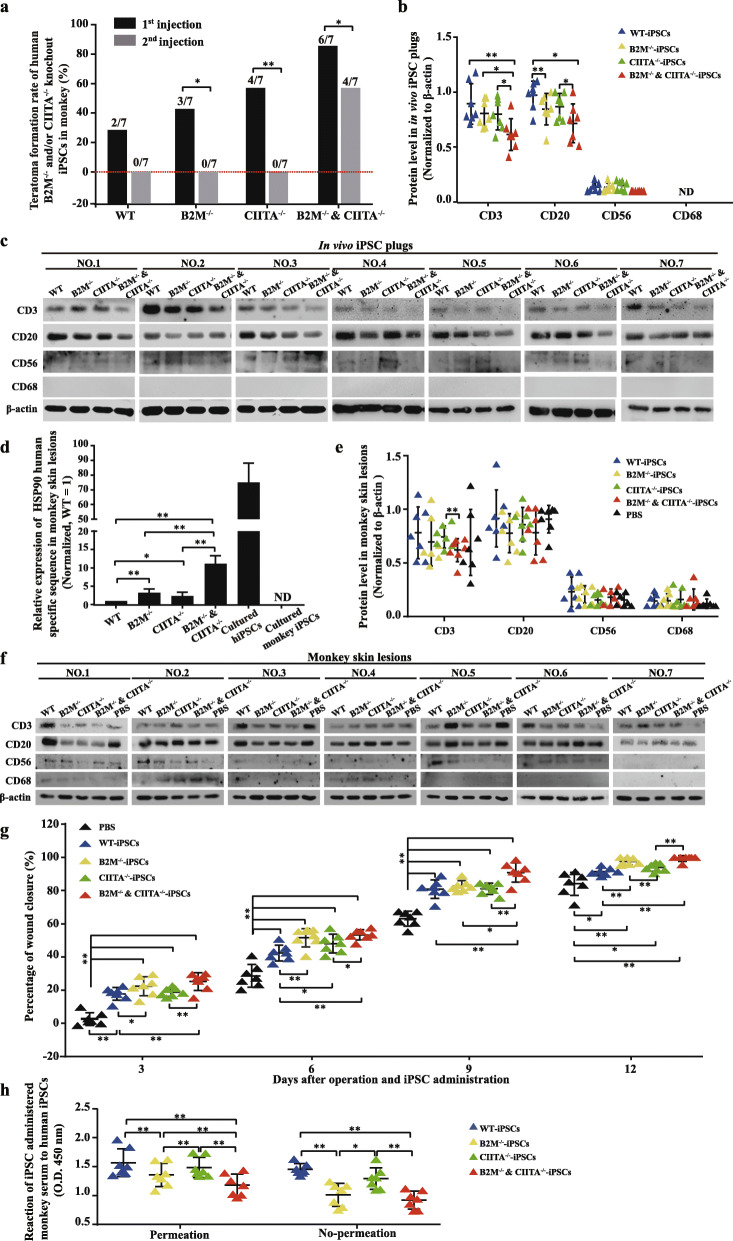
Teratoma formation, in vivo survival, effects on infiltration of immune cells, and wound healing promotion of knock-out iPS cell lines. **a** Teratoma generation rates of human WT-iPSCs, B2M^−/−^-iPSCs, CIITA^−/−^-iPSCs, and B2M^−/−^ and CIITA^−/−^-iPSCs in monkeys. **b**, **c** Gray scale statistics and images of Western blotting show the presence of CD3, CD20, CD56, and CD68 proteins in the tissue 3 days after Matrigel-embedded human iPSCs were subcutaneously injected into the monkey. **d** Relative expression levels of human-specific HSP90 sequence demonstrate the survival of transplanted human iPSCs in monkey wound lesions. **e**, **f** Gray scale statistics and images of Western blotting show the presence of CD3, CD20, CD56, and CD68 proteins in monkey wound lesions 12 days after human iPSCs were topically disseminated onto the surface of fresh lesions. **g** Wound closure was measured for 12 days and percentages of wound closure are presented. Data represent the means ± SD. **h** Immune response of different cell lines to monkey serum measured by ELISA. Data represent the means ± SD; *n* = 7 in all measurements. Data in **a** was evaluated by chi-square test. All other data sets were evaluated by paired Student’s *t* test

The ability of engineered human iPSCs to form tumor mass in monkeys was observed using classic teratoma generation assay by embedding the cells in extracellular matrix (Matrigel) before the cells were injecting subcutaneously [22]. Tumor mass formation rate within 18 days of the first injection of wild type (WT), B2M^−/−^, CIITA^−/−^, and B2M^−/−^ and CIITA^−/−^ were 2/7, 3/7, 4/7, and 6/7, respectively. The rate after second injection decreased to 0/7, 0/7, 0/7, and 4/7 (Fig. 5a, Supplemental Table 2). Thus, both of B2M and CIITA contributed to the incapability of forming tumor-like mass. However, all the tumors became non-detectable within 28 days. All the 4 human cell lines generated teratoma in SCID-NOD immune-deficient mice. However, none of them formed teratoma in the immune-competent C57 mice (Supplemental Table 3 and Supplemental Fig. 1, *n* = 10).

### B2M and CIITA knockout decreases the immune response of monkey recipients to administered human iPSCs in vivo

To observe immune response against the cells, the cell plugs (cumulated in Matrigel) were harvested 3 days after third administration (at different location each time, of course) in monkeys. Western blotting found dual knockouts of B2M and CIITA significantly decrease CD3 and CD20 amounts in the cell plugs compared to that in WT iPSC plugs. Single B2M knockout decreased CD20, but not CD3; single CIITA knockout affected neither CD20 nor CD3. CD56 (a marker of NK cells) amount had no difference across the cell plugs. CD68 was not detectable at all (Fig. 5b, c).

### B2M and CIITA knockout increases survival and decreases the immune response to disseminated human iPSCs in monkey skin wounds

To observe the immune response to disseminated human iPSCs in monkey, human iPSCs were spread onto newly created monkey skin wounds and harvested along with skin wounds 12 days after iPSC application. Survival of the human iPSCs in monkey skin wound lesions was shown by human heat shock protein 90 (HSP90) using specific target primers targeting to human but not to monkey HSP90. Specificity of the primers was confirmed by PCR assay of the cultured human and monkey iPSCs. The survival rates of B2M^−/−^, CIITA^−/−^, and B2M^−/−^ and CIITA^−/−^ were 3.24, 2.31, and 11.17 times that of WT human iPSCs (Fig. 5d). CD3 amount in lesions treated with B2M^−/−^ and CIITA^−/−^ was significantly less than in lesions treated with CIITA^−/−^. However, CD3 was not significantly different among other groups. CD20, CD56, and CD68 were not significantly different across the five groups, including the lesions without iPSC treatment (Fig. 5e, f). Thus, disseminated iPSCs were less susceptible to immune rejection than cumulated iPSCs.

### B2M and CIITA knockout increases the pro-wound healing properties of disseminated human iPSCs in monkeys

The therapeutic effect of corresponding topically administered iPSCs was determined by observing the percentage of wound closure every 3 days for 12 days. All human WT, B2M^−/−^, CIITA^−/−^, and B2M^−/−^ and CIITA^−/−^ cells promoted wound healing in monkey (Fig. 5g and Supplemental Fig. 2). iPSCs with single knockout were better than those without a knockout. Cells of dual knockouts were better than single knockout.

### Either B2M or CIITA knockout inhibits iPSC-dependent stimulation of allogenic T lymphocytes and increases iPSC-differentiated CD3-positive cells

**Fig. 6:**
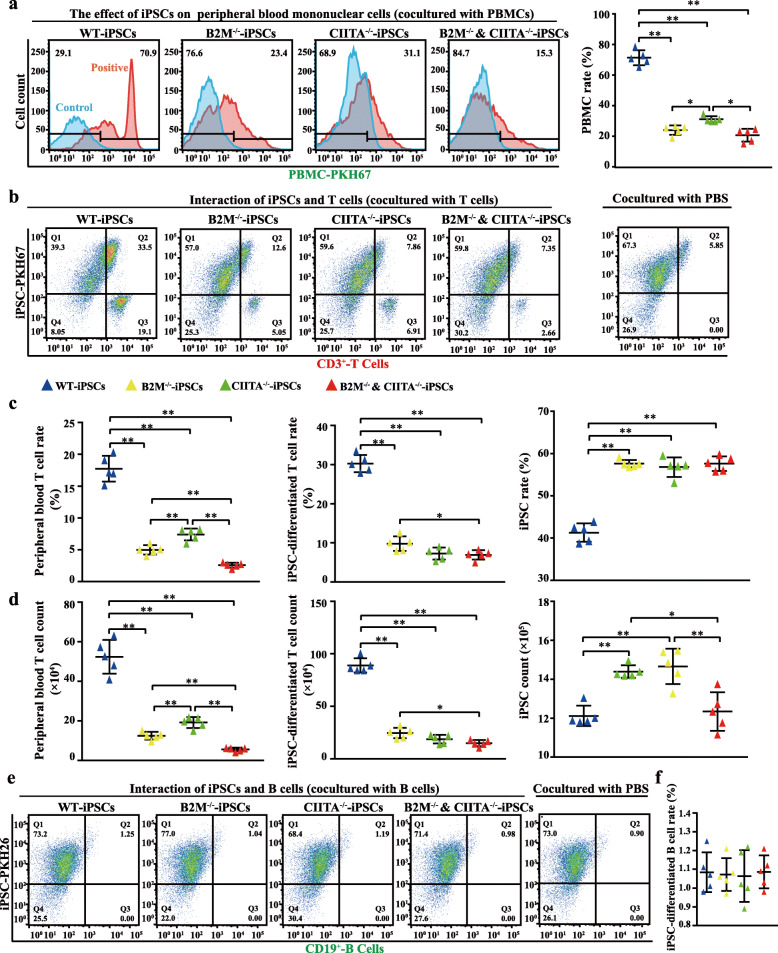
Effects of B2M and/or CIITA on the ability of iPSCs to promote proliferation of allogeneic human circulatory immune cells. **a** Human peripheral blood mononucleated cells were labeled with a green fluorescent dye and added onto the different iPS cell lines. Green fluorescence-positive cells were counted by flow cytometry after co-incubation for 7 days. Percentage of the positive cells is presented. **b**, **d** Red fluorescence-labeled CD3-positive peripheral T lymphocytes were co-incubated with four different iPS cell lines (labeled with a green fluorescent dye) for 7 days. CD3-positive cells, which included previously added CD3-positive lymphocytes and possible iPSC-derived CD3-positive cells in mixed cells, was then labeled with red fluorescence followed by flow cytometry (**b**). Percentages (**c**) and number counts (**d**) of red, green, and double-positive cells (representing peripheral blood T lymphocytes, non-differentiated iPSCs and iPSC-derived CD-3 positive cells) are presented. **e**, **f** Green fluorescence-labeled CD19-positive cells were added onto the different iPS cell lines (labeled with a red fluorescent dye) for co-culture. After co-incubation for 7 days, CD19-positive cells in the mixed cells were labeled with green fluorescence. Green and red fluorescence-positive cells were then counted by flow cytometry. Flow cytometry chart and statistical result are shown. Data represent the means ± SD; *n* = 5 in **a**–**f** panels. Data sets in **a**–**f** were evaluated by paired Student’s *t* test

To confirm this assumption, we examined the stimulatory effect of B2M and/or CIITA knockout on allogeneic PBMCs, T lymphocytes, or B lymphocytes from 5 different healthy individuals. To prevent cross immune-recognition and response, each cell line was co-incubated separately with cells from each person. The cells were labeled with different fluorescent dyes and co-incubated separately with the allogeneic blood cells for 7 days in lymphocyte culture medium and eventually counted by flow cytometry. Either B2M or CIITA knockout was found to inhibit the stimulatory effect of iPSCs on PBMC proliferation, with B2M and CIITA dual knockout diminishing this effect the most (Fig. 6a).

The response of isolated T lymphocytes to iPSCs of B2M and/or CIITA knockout was similar to that of PBMCs. Some iPSCs in the co-culture system became CD3-positive meanwhile. The rate of iPSC-derived CD3-positive cells decreased from 30.28 ± 2.21% in WT to 9.80 ± 1.89%, 7.26 ± 1.55%, and 6.92 ± 1.19% in B2M^−/−^, CIITA^−/−^, and B2M^−/−^ and CIITA^−/−^, respectively. Accordingly, the rate of iPSCs in the co-cultured cell population decreased (Fig. 6b, c). The count of different cells affirmed the change in percentage (Fig. 6d). This in vitro result was consistent with the in-vivo results in the human-to-monkey study.

In contrast to T lymphocytes, none of WT, B2M^−/−^, CIITA^−/−^, and B2M^−/−^ and CIITA^−/−^ stimulated the proliferation of peripheral blood B lymphocytes or differentiation into cells expressing B lymphocyte marker within 7 days of co-incubation in lymphocyte-culturing medium (Fig. 6e, f).

### The seropositivity of monkey serum in response to human iPSCs is reduced following B2M and CIITA knockout

Measured by ELISA, all four iPS cell lines showed positive reactivity to the sera harvested from monkeys after the last (fourth) iPSC administration (Supplemental Fig. 3). Without cell-membrane permeation (for measurement of extracellular episodes), the reactivity of B2M^−/−^ and B2M^−/−^ and CIITA^−/−^ to serum was significantly lower than that of WT. CIITA knockout alone did not have significant effect on reactivity. With cell-membrane permeation, although the reactivity did not differ between CIITA^−/−^ and WT, that of B2M^−/−^ and CIITA^−/−^ was significantly lower than in CIITA^−/−^or B2M^−/−^ alone (Fig. 5h).

## Discussion

The future of stem cell therapy is mainly dependent on overcoming the side-effects, rather than finding their new therapeutic potentials. We had previously hypothesized that if iPSCs were disseminated in disease lesions, they would be controlled by tissue microenvironment and behave based on the needs of local regeneration. In 53 experimental conditions and animal models including human, monkey, and mouse, we demonstrated that disseminated isogeneic and autologous iPSCs did not form teratoma or quiesce in vivo [20]. In this study, we knocked out human B2M and CIITA in human iPSCs either separately or simultaneously, and this did not affect iPSC pluripotency. The knockouts diminished the expression of MHC alleles and minimized immunogenicity of the dual knockout cells for allogeneic human lymphocytes in vitro and for rhesus macaque recipients in vivo. Dual knockout human iPSCs were optimal in terms of their reduced immunogenicity, their increased survival, and their ability to accelerate wound healing in monkeys. We have previously demonstrated that iPSCs transplanted onto skin wounds in monkeys accelerate the wound healing by promoting epithelial recovery, angiogenesis, and collagen deposition through paracrine signaling and differentiating into endothelial cells. Further, autologous monkey iPSCs were more effective than allogeneic iPSCs in promoting would healing [6]. Taken together, our results demonstrate that reducing the immunogenicity of allogeneic (and, in our case, even xenogenic) stem cells can improve stem cell therapy.

MHC I contains α and β subunits. B2M is the β subunit. However, the binding site of NK inhibitory receptor is located in the α subunit [23]. Our results showed that the expression of α subunit was not completely inhibited upon knocking out B2M. The mRNA levels of HLA-A, HLA-B, and HLA-C α subunits were decreased only by 43.3, 72.7, and 46.3%, respectively. However, downregulation of mRNA levels does not necessarily translate into decreased protein levels. Although the mechanism underlying the B2M-knockout-induced downregulation of α subunit mRNAs is elusive, there may be a reciprocal regulation between α and β subunits since the two subunits assemble in 1:1 ratio to form MHC I. For example, the absence of β subunit may induce the degradation of α subunit or cause an inhibitory feedback response on the transcription of α subunit. Nevertheless, the remaining α subunits may be sufficient for NK inhibition. In addition, difference in CRISPR-Cas9 editing site of B2M gene may cause different HLA expression and deformation of the NK binding structure, and hence different NK recognition. Besides, the process attracting circulatory NK cells to the administered iPSCs in lesions is complicated. Our results did not show any difference in this respect across the four cell lines. In fact, to avoid long-term carcinogenic mutation (not unwanted benign differentiation such as forming teratoma) in vivo, we did not want our cells to be eternal and completely avoid the recipient’s immune system. Optimized knockout sites of B2M may produce iPSCs that meet the therapeutic needs better, in addition to feasibility regarding time, cost, standardization, and workload. The fact that we found iPSCs may differentiate into CD3-positive cells indicated that the cells might traffic to, reside, and differentiate into immune cells in bone marrow, if administered in vivo. This provided another reason for the cells to not be eternal in vivo, in order to avoid graft-versus-host disease.

It was also worth to mention that, although undifferentiated iPSCs did not express CIITA, B2M^−/−^ and CIITA^−/−^ were more resistant to immune attack and less stimulatory to immune cells than B2M^−/−^ single knockout. This could be attributed to CIITA being a regulator [15], and not MHC II itself. It might have functions other than regulating the expression of MHC II.

The positive therapeutic result in wound healing indicated the feasibility of using our engineered cells in repeated administration because this is the fourth delivering of the cells in the monkeys. Since human B2M^−/−^ and CIITA^−/−^ formed tissue mass in monkeys, but not in mice, species similarity may play an important role. Human B2M^−/−^ and CIITA^−/−^ may have better therapeutic effect in allogeneic humans for two reasons. First, although non-human primates are closest to humans, human iPSCs are xenogeneic to monkeys. The immune histocompatibility is more similar between allogeneic humans than between monkey and human. Second, since each monkey had received all four cell types, the administered iPSCs had to bear immune attack stimulated by all four, although to different extents due to different recognizabilities.

Since the monkeys had received all four types of cells simultaneously, the in vivo measurement could only determine the reactivity to activated immune system, and not identify which cell lines contribute to immune activation and what is the extent of its contribution. Since the similarity of antigens initiates antibody production and binding, we believed B2M^−/−^ and CIITA^−/−^ to be the cell line least capable of stimulating immune response in monkey. This assumption was confirmed by our in vitro study, which showed that either B2M or CIITA knockout inhibited the stimulatory effect of iPSCs on the proliferation of allogeneic PBMC and T lymphocytes isolated from healthy individuals. We note that dual knockout of B2M and CIITA had a greater effect than either of the single knockouts.

## Conclusions

In summary, B2M-plus-CIITA knockout of human iPSCs diminished immune response while promoting survival and therapeutic effects in monkeys. These beneficial outcomes may be more significant in allogeneic humans, since species similarity has a high impact. The survival of transplanted iPSCs with B2M-plus-CIITA knockout may be similar to that in HLA-closely matched allografts. Our study would therefore help to remove the bottlenecks that prevent most iPSC benefits from real clinical application.

## Supplementary information

**Additional file 1: Figure S1.** Teratoma formation of the human knockout iPSC lines in mice. iPSCs were subcutaneously injected into NOD-SCID mice and C57BL/6 mice. All the knockout cells formed teratoma in SCID-NOD immune-deficient mice in two months after injection but not in the immune-competent C57 mice in six months after injection.

**Additional file 2: Figure S2**. (Related to Fig. 5) Promotion of wound healing by knockout-iPSCs. Representative images of wounds treated with PBS, WT-iPSCs, B2M^−/−^-iPSCs, CIITA^−/−^-iPSCs, and B2M^−/−^& CIITA^−/−^-iPSCs on 0, 3, 6, 9, and 12 days after wound punching, followed immediately by iPSC treatment.

**Additional file 3: Figure S3.** Timeline of the study.

**Additional file 4: Supplemental Table 1.** Anti-human iPSC antibodies in monkey serum.

**Additional file 5: Supplemental Table 2-3.** Summaries of teratoma formation rates from human iPSCs in monkey and mouse.

## Data Availability

All raw data generated or analyzed in this study are available at: http://homepage.fudan.edu.cn/xiangmeng/stem cell research and therapy/. Protocols for the methods used in this study are available from the corresponding author upon request.

## Abbreviations

iPSCs: Induced pluripotent stem cells
MHC: Major histocompatibility complex
B2M: Beta2 microglobulin
CIITA: Major histocompatibility complex class II transactivator
HLA: Human leukocyte antigen
AP: Alkaline phosphatase
EB: Embryoid body
DMEM/F12: Dulbecco’s modified Eagle’s medium/nutrient mixture F-12 medium
KSR: Knockout serum replacement
NEAA: Non-essential amino acids solution
2-ME: 2-Mercaptoethanol
Oct3/4: Octamer-binding protein 3/4
Sox2: SRY-Box transcription factor 2
SSEA-4: Stage-specific embryonic antigen-4
DAPI: 4′,6-Diamidino-2-phenylindole
ELISA: Enzyme-linked immunosorbent assay
PBMCs: Peripheral blood mononuclear cells
PBS: Phosphate buffer saline
