# Supplementary figures and images for: Diminished expression of major histocompatibility complex facilitates the use of human induced pluripotent stem cells in monkey
Source: Stem Cell Res Ther. 2020 Aug 3;11:334. doi: 10.1186/s13287-020-01847-9 (PMC7397609; doi:10.1186/s13287-020-01847-9)

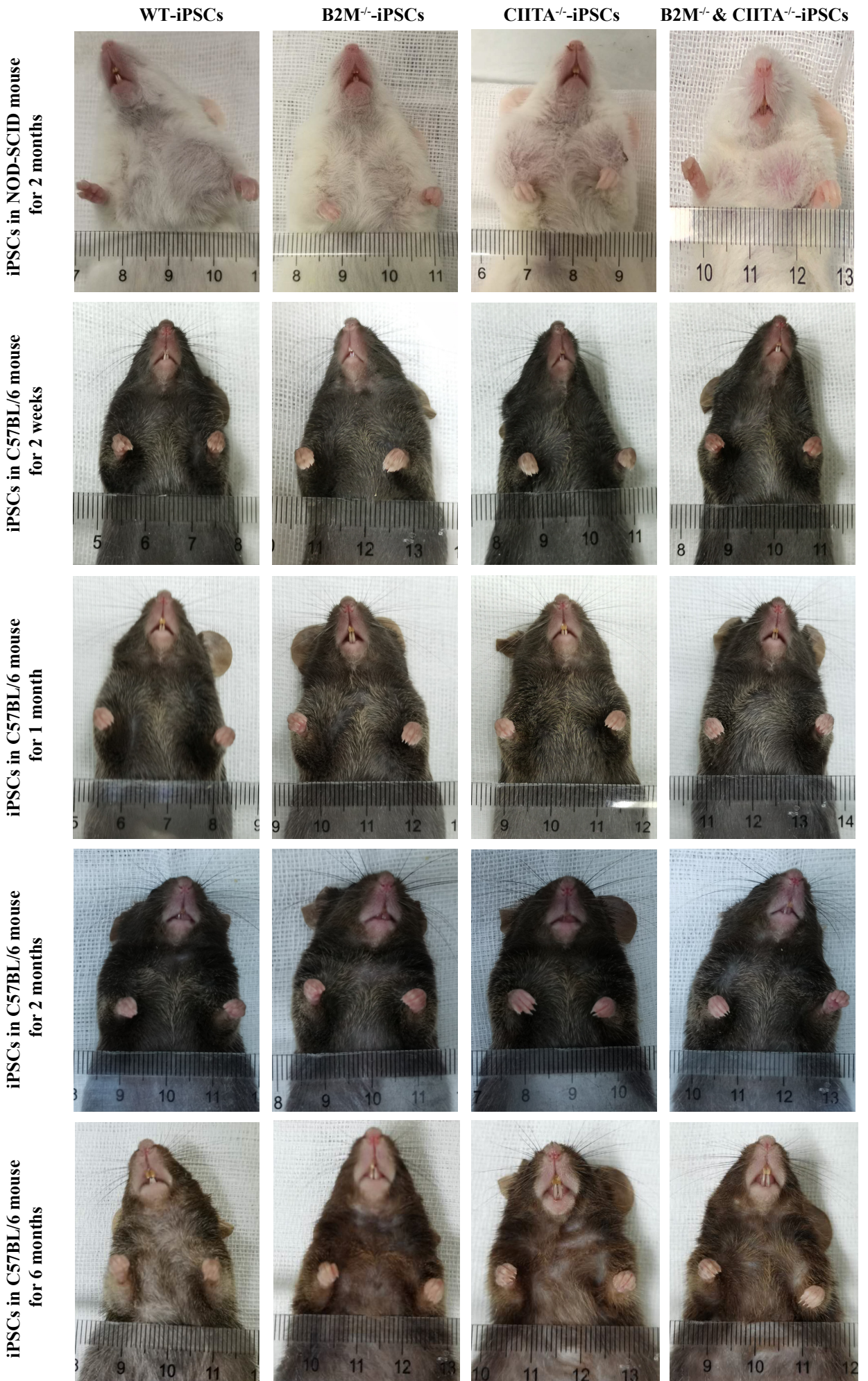

Figure S1. Teratoma formation of knock-out iPS cell lines in mice.

Supplement: Supplementary file 1 — Additional file 1: Figure S1. Teratoma formation of the human knockout iPSC lines in mice. iPSCs were subcutaneously injected into NOD-SCID mice and C57BL/6 mice. All the knockout cells formed teratoma in SCID-NOD immune-deficient mice in two months after injection but not in the immune-competent C57 mice in six months after injection. [file 13287_2020_1847_MOESM1_ESM.pdf]

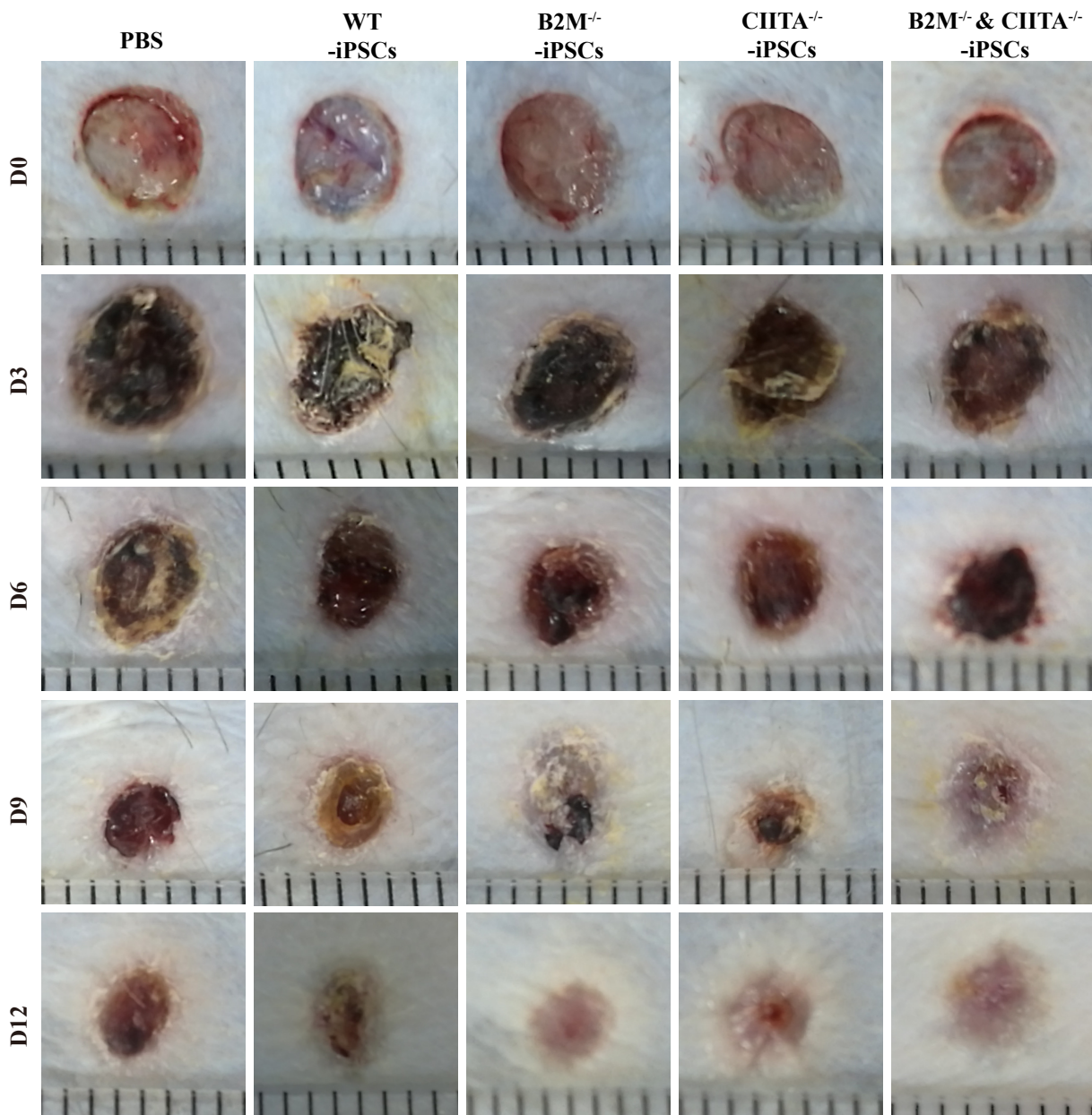

**Figure S2. Related to Fig. 5 Knockout iPSCs promote wound healing.**

Supplement: Supplementary file 2 — Additional file 2: Figure S2. (Related to Fig. 5) Promotion of wound healing by knockout-iPSCs. Representative images of wounds treated with PBS, WT-iPSCs, B2M−/−-iPSCs, CIITA−/−-iPSCs, and B2M−/−& CIITA−/−-iPSCs on 0, 3, 6, 9, and 12 days after wound punching, followed immediately by iPSC treatment. [file 13287_2020_1847_MOESM2_ESM.pdf]

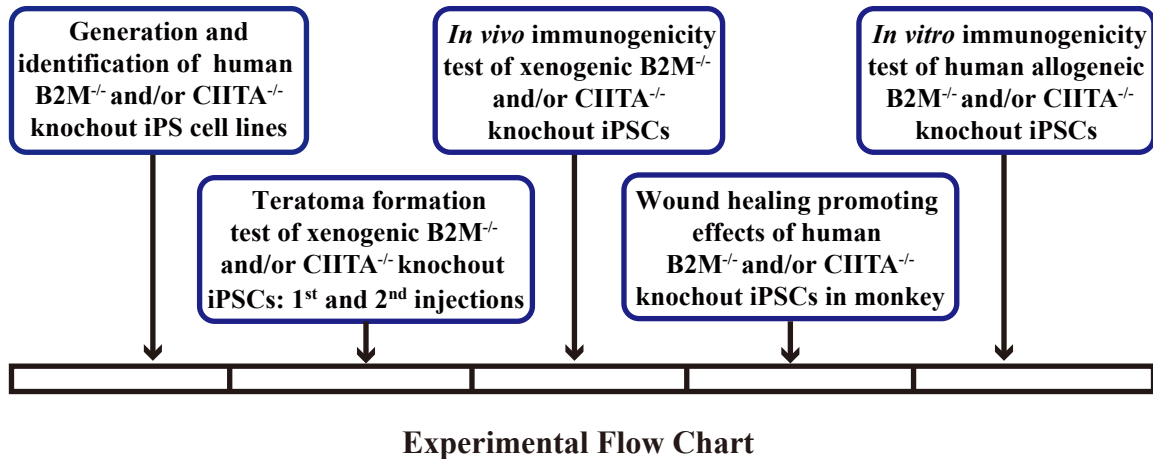

**Figure S3. Time line of the study.**

Supplement: Supplementary file 3 — Additional file 3: Figure S3. Timeline of the study. [file 13287_2020_1847_MOESM3_ESM.pdf]
