# Supplementary material for: Diminished expression of major histocompatibility complex facilitates the use of human induced pluripotent stem cells in monkey
Source: Stem Cell Res Ther. 2020 Aug 3;11:334. doi: 10.1186/s13287-020-01847-9 (PMC7397609; doi:10.1186/s13287-020-01847-9)
Supplement: Supplementary file 4 — Additional file 4: Supplemental Table 1. Anti-human iPSC antibodies in monkey serum. [file 13287_2020_1847_MOESM4_ESM.pdf]

Supplemental Table1. Anti-human iPSC antibodies in monkey serum

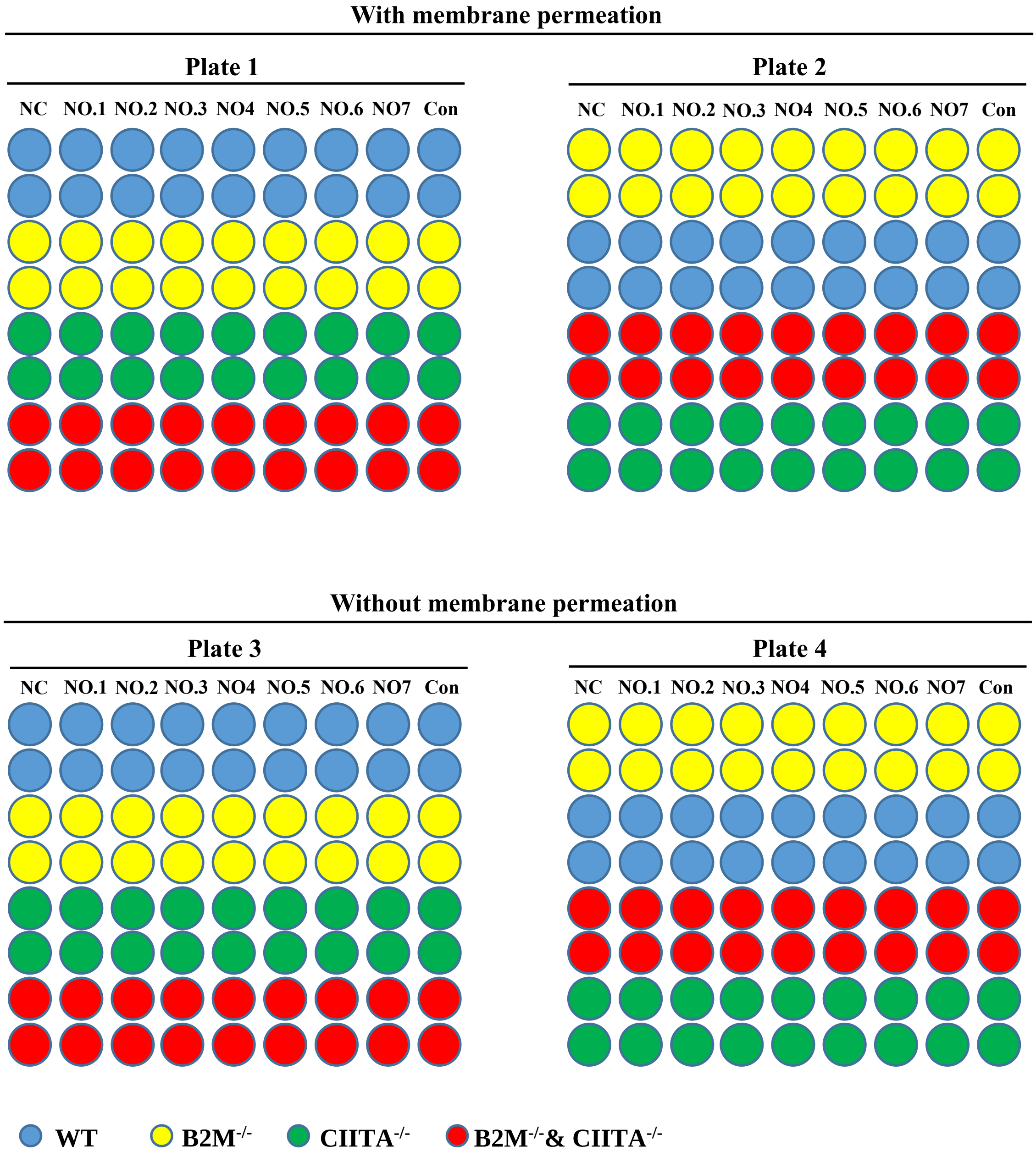

NC: negative control, using PBS instead of monkey serum;

No.: number of monkeys;

Control: using serum from normal monkey that did not receive iPSCs
