# Supplementary material for: Diminished expression of major histocompatibility complex facilitates the use of human induced pluripotent stem cells in monkey
Source: Stem Cell Res Ther. 2020 Aug 3;11:334. doi: 10.1186/s13287-020-01847-9 (PMC7397609; doi:10.1186/s13287-020-01847-9)
Supplement: Supplementary file 5 — Additional file 5: Supplemental Table 2-3. Summaries of teratoma formation rates from human iPSCs in monkey and mouse. [file 13287_2020_1847_MOESM5_ESM.pdf]

Supplemental Table2.

A Summary of Teratoma Formation Rate from human iPSCs in Monkey								
**NO.**	**WT-iPSCs**		**B2M$^{-/-}$-iPSCs**		**CIITA$^{-/-}$-iPSCs**		**B2M$^{-/-}$ & CIITA$^{-/-}$-iPSCs**	
	**1st**	**2nd**	**1st**	**2nd**	**1st**	**2nd**	**1st**	**2nd**
**NO.1**	0/1	0/1	0/1	0/1	0/1	0/1	1/1	1/1
**NO.2**	1/1	0/1	1/1	0/1	1/1	0/1	1/1	0/1
**NO.3**	0/1	0/1	1/1	0/1	1/1	0/1	1/1	1/1
**NO.4**	0/1	0/1	0/1	0/1	0/1	0/1	1/1	1/1
**NO.5**	0/1	0/1	0/1	0/1	0/1	0/1	0/1	0/1
**NO.6**	0/1	0/1	0/1	0/1	1/1	0/1	1/1	0/1
**NO.7**	1/1	0/1	1/1	0/1	1/1	0/1	1/1	1/1
**Total**	**2/7**	**0/7**	**3/7**	**0/7**	**4/7**	**0/7**	**6/7**	**4/7**

Supplemental Table3.

A Summary of Teratoma Formation Rate from human iPSCs in Mouse				
**NO. a-d**	**WT-iPSCs(a)**	**B2M$^{-/-}$-iPSCs (b)**	**CIITA$^{-/-}$-iPSCs (c)**	**B2M$^{-/-}$ & CIITA$^{-/-}$-iPSCs(d)**
**1**	0/1	0/1	0/1	0/1
**2**	0/1	0/1	0/1	0/1
**3**	0/1	0/1	0/1	0/1
**4**	0/1	0/1	0/1	0/1
**5**	0/1	0/1	0/1	0/1
**6**	0/1	0/1	0/1	0/1
**7**	0/1	0/1	0/1	0/1
**8**	0/1	0/1	0/1	0/1
**9**	0/1	0/1	0/1	0/1
**10**	0/1	0/1	0/1	0/1
**Total**	**0/10**	**0/10**	**0/10**	**0/10**
